# Perceived Neighborhood Social Environment and Adolescent Depressive Symptoms: Insights from the Add Health

**DOI:** 10.1089/heq.2024.0100

**Published:** 2024-12-16

**Authors:** Rachel J. Kulchar, Breanna J. Rogers, Sam J. Neally, Alyssa Shishkov, Yangyang Deng, Mohammad Moniruzzaman, Kosuke Tamura

**Affiliations:** ^1^ Department of Chemistry, Princeton University, Princeton, New Jersey, USA.; ^2^ Socio-Spatial Determinants of Health (SSDH) Laboratory, Population and Community Health Sciences Branch, Division of Intramural Research, National Institute on Minority Health and Health Disparities, National Institutes of Health, Bethesda, Maryland, USA.; ^3^ Gillings School of Public Health, University of North Carolina, Chapel Hill, North Carolina, USA.; ^4^ University of California Santa Barbara, Santa Barbara, California, USA.

**Keywords:** neighborhoods, social contexts, mood disorders, depression, youth, disparities

## Abstract

**Introduction:**

Adolescents experience major depression at disproportionately higher rates than their adult counterparts. Perceived neighborhood social environment (PNSE) has been linked with depressive symptoms among adolescents. The primary aim was to investigate the relationships between each PNSE and depressive symptoms. The secondary aim was to examine whether these associations may be varied by gender and race/ethnicity.

**Methods:**

Participants (*n* = 6083; mean age = 15.4) from the 1994–1995 National Longitudinal Study of Adolescent to Adult Health (Add Health) were asked to respond to items on depressive symptoms using the Center for Epidemiologic Studies Depression Scale (CES-D) and perceived neighborhood measures. The two depressive symptoms outcomes based on CES-D score were a continuous CES-D score and a three-level depressive symptoms variable: (i) minimal symptoms score (referent) <16, (ii) mild: 16 ≤ CES-D < 24, and (iii) moderate/severe: CES-D ≥24. PNSE included safety, social cohesion, and contentedness (i.e., 1-standard deviation unit increase). Weighted regression models were used to examine associations between each PNSE and depressive symptoms, adjusting for covariates.

**Results:**

Perceived neighborhood safety, social cohesion, and contentedness were negatively related to depressive symptoms (β = −1.14, β = −0.59, and β = −1.46, respectively, all *p* < 0.001). Similar patterns of negative associations were observed by gender, whereas race/ethnicity-specific analyses revealed the complexity of the associations.

**Conclusion:**

As adolescents’ favorable perceptions of their neighborhoods (safety, social cohesion, and contentedness) were related to lower depressive symptoms, efforts toward improving neighborhood conditions and resources may be imperative to drive health equity in specific subgroups and address disparities in the adolescent mental health epidemic.

## Introduction

Major depression is defined as a disorder persisting for at least 2 weeks wherein individuals report experiencing negative affect, anhedonia, and problems sleeping, eating, and concentrating.^1–3^ Before mental health concerns worsened during the COVID-19 pandemic, mental health problems were the primary drivers of disability and death among adolescents.^4^ Moreover, major depressive disorder (MDD) among youth has been identified as a risk factor for chronic diseases, such as cardiovascular disease.^5,6^ The prevalence of major depressive episodes (MDE) is high among adolescents, with those ages 12–17 experiencing MDE at nearly 2.5 times higher prevalence rates than their adult counterparts.^3^

In 2021, more than one in five adolescents living in the United States (U.S.) experienced an MDE, disproportionately impacting certain groups. Female adolescents are almost 2.5-fold more likely to experience an MDE than male counterparts.^3^ A study found a significant increase in MDE prevalence later in life, especially among females, highlighting the need to better understand the risk and protective factors associated with depression among adolescents.^7^ Moreover, over the past decade, there has been an increase in MDEs observed among non-Hispanic (NH) White, Black, and Hispanic adolescents alike.^4^ Depression estimates were highest in Hispanic adolescents (22.2%), followed by White (20.7%), Black (14.0%), and Asian (13.8%) adolescents.^3^ However, due to misdiagnosis and underdiagnosis among minority populations, some of these estimates are likely underestimated.^8,9^

It has been widely recognized that MDE onset is compounded by environmental determinants, especially among adolescents.^10–12^ For example, some studies indicate that perceived neighborhood social cohesion (trusting neighbors) and structural disadvantage (inadequate availability of material, institutional, and social support) were negatively and positively associated with depression, respectively.^11,13^ Another study consisting of nearly 4,500 Californian adolescents found that those with higher perceived neighborhood safety were two-fold less likely to disclose feelings of serious psychological distress than those with lower perceived neighborhood safety.^14^

Few studies have investigated the association between perceived neighborhood social environment (PNSE) and depression among adolescents as influenced by gender and race/ethnicity. A recent study of 1,200 Black adolescents indicated that gender and race/ethnicity could play a role in how perceived neighborhood environments are related to the onset of MDE.^15^ However, this study explored this relationship only among Black adolescents. Results indicated that, unlike African American males, those perceiving unsafe neighborhood environments had higher MDD scores among Caribbean Black males and Caribbean Black or African American females.^15^ Therefore, only a few studies have sought to better understand the relationship between PNSE and depressive symptoms among U.S. adolescents and examined differential associations by gender and race/ethnicity. The primary aim of this study was to examine the association between PNSEs (i.e., safety, social cohesion, and contentedness) and depressive symptoms. The exploratory aim was to test whether the associations may be varied by gender and race/ethnicity separately.

## Materials and Methods

### Data and Study Participants

This study conducted secondary data analyses of the National Longitudinal Study of Adolescent to Adult Health (Add Health). Add Health is a nationally representative longitudinal study that includes responses from over 20,000 adolescents ages 12–19. Since the study began in 1994, five follow-up interviews have been conducted.^16^ This cross-sectional study used data from Wave I, which was collected in 1994–1995. Out of the 20,000 adolescents who participated in Add Health, survey weights were only included for 6,504 adolescents. Of these 6,504 adolescents, 13 were excluded due to missing depression measures, and 30 were removed as they were 20 years old and older. Furthermore, 188 were excluded due to missing race/ethnicity and/or parental education responses. Participants who missed responses related to neighborhood perceptions (*n* = 190) were also excluded, resulting in a total sample of 6,083. Add Health received ethical approval for all aspects of its protocol from the University of North Carolina at Chapel Hill IRB.

### Depressive Symptoms

Add Health includes a variety of questions pertaining to adolescent depression. The Add Health study adapted the Center for Epidemiologic Studies Depression Scale (CES-D) to include 19 items as opposed to 20 items.^17,18^ Depressive symptoms among adolescents were analyzed through these 19 items from the CES-D^18,19^ by producing an average score from the 19 items. This value was then multiplied by 20 to confirm reproducibility with the standard 20-item CES-D.^18^ These items were composed of questions that assessed how often an adolescent felt depressive symptoms on a weekly basis and were provided a score from zero (“never”) to three (“daily”) based on such frequency. Although the CES-D was constructed to investigate depressive symptoms among adults, it has also been validated among adolescents.^20^ A CES-D score <16 represents “minimal” depressive symptoms (referent group). A CES-D score of 16 ≤ CES-*D* < 24 represents “mild” symptoms.^20,21^ A CES-D score ≥24 is indicative of “moderate/severe” depressive symptoms.

### Perceived Neighborhood Social Environment

To assess how adolescents viewed their neighborhood environment, PNSE factors were assessed. Three PNSE factors included neighborhood (i) safety, (ii) social cohesion, and (iii) contentedness. Specifics pertaining to these three PNSE constructs were previously reported. Safety included one item and assessed how safe the adolescent felt in their neighborhood. Perceptions of social cohesion were assessed through three metrics: (i) willingness to help neighbors, (ii) trust in neighbors, and (iii) overall friendliness of neighbors. Each score was summed to create a social cohesion factor. Contentedness was measured as an overall sum of scores associated with neighborhood safety, social cohesion, collective efficacy (“Do people in your neighborhood stick up for each other?”), and happiness (“If, for any reason, you had to move from there to some other neighborhood, how happy or unhappy would you be?”).^22^ Each PNSE was transformed into the standard deviation (SD) from the mean (i.e., 1-SD unit increase) to ease the interpretation.

### Covariates

Consistent with previous studies, covariates could confound the relationship between PNSEs and depressive symptoms, which were included in the analyses.^20^ Adolescent demographic characteristics included age (in years), gender (female and male), and race/ethnicity (NH White, NH Black, Hispanic, and other [Native American, Asian or Pacific Islander, or other] adolescents). Factors related to parental socioeconomic position include parent’s educational attainment (i.e., less than high school, high school, some college, and college or more). Adolescent health-related covariates consisted of the total physical activity (PA) level and body mass index (BMI). Finally, neighborhood poverty (low, medium, and high) at the census block group level, which could confound other neighborhood characteristics, was also controlled for.

### Statistical Analysis

Weighted means and associated standard errors of a continuous variable and the weighted frequencies with percentages of categorical variables were used for descriptive statistics (Table 1) and stratified by depressive symptoms (minimal, mild, and moderate/severe). To assess the association between PNSE and depressive symptoms, weighted linear regression models were used in the analyses for a continuous depressive symptoms outcome. By accounting for a multinomial outcome, weighted logistic regression models (Proc survey logistic) for odds ratios (OR) with 95% confidence intervals (95% CI) were used in the multinomial depressive symptoms outcome (minimal [referent], mild, and moderate/severe). Gender-specific and race/ethnicity-specific associations were analyzed separately. All analyses were adjusted for covariates and conducted using SAS 9.4 (SAS, Cary, NC).^23^

**Table 1.** Participants’ Characteristics and Stratified by Severity of Depressive Symptoms (*n* = 6,083)

		Depression symptoms (score^a^)		
	Overall (*n* = 6,083)	Minimal symptoms (CES-D < 16; *n* = 4,723; 0)	Mild symptoms (16 ≤ CES-D < 24; *n* = 907; 1)	Moderate/severe (CES-D ≥ 24; *n* = 453; 2)
Age, mean (SEM)^b^	15.42 (0.03)	15.33 (0.03)	15.75 (0.06)	15.71 (0.09)
Sex, *n* (%)				
Male	2,941 (50.61)	2,421 (53.45)	371 (42.81)	149 (36.20)
Female	3,142 (49.39)	2,302 (46.55)	536 (57.19)	304 (63.80)
Race/ethnicity, *n* (%)				
Non-Hispanic White	3,558 (66.48)	2,884 (69.30)	453 (57.11)	221 (55.49)
Non-Hispanic Black	1,455 (15.49)	1,087 (14.09)	254 (21.20)	114 (18.86)
Hispanic	675 (11.89)	478 (10.94)	127 (14.58)	70 (16.55)
Others^*^c^*^	395 (6.14)	274 (5.67)	73 (7.11)	48 (9.10)
Parent education, *n* (%)				
Less than high school	552 (9.93)	365 (8.49)	109 (13.13)	78 (18.74)
High school	1,814 (31.92)	1,342 (30.71)	311 (35.31)	161 (37.87)
Some college	1,255 (20.65)	1,002 (21.13)	185 (22.04)	68 (12.91)
College or more	2,462 (37.50)	2,014 (39.67)	302 (29.53)	146 (30.47)
Neighborhood poverty, *n* (%)				
High	1,343 (20.10)	989 (18.82)	240 (24.79)	114 (24.19)
Medium	1,390 (23.53)	1,073 (23.35)	204 (23.71)	113 (25.11)
Low	3,350 (56.37)	2,661 (57.83)	463 (51.50)	226 (50.70)
MVPA, mean (SEM)^d^	6.02 (0.05)			
BMI, mean (SEM)^e^	24.54 (0.27)	24.51 (0.31)	25.28 (0.74)	23.51 (0.66)
Depressive symptoms, mean (SEM)	10.81 (0.11)	7.66 (0.07)	18.50 (0.07)	28.61 (0.26)
Perceived neighborhood social environments, mean (SEM)				
* *Safety	0.90 (0.00)	0.92 (0.00)	0.83 (0.01)	0.75 (0.02)
* *Social cohesion	2.28 (0.01)	2.33 (0.02)	2.11 (0.04)	2.09 (0.05)
* *Contentedness	6.86 (0.03)	7.07 (0.03)	6.25 (0.07)	5.88 (0.13)

^a^ CES-D, Center for Epidemiologic Studies Depression Scale.

^b^ SEM, standard error of mean.

^c^ Others include Native American, Asian or Pacific Islander, or others.

^d^ MVPA, moderate-to-vigorous physical activity.

^e^ BMI, body mass index.

## Results

### Descriptive Statistics

The present study includes data from 6,083 adolescents, with around half being of the male gender (Table 1). The mean age of the participants was 15.4 years old (standard error of mean [SEM] ± 0.03). Most participants identified as NH White (66.5%). Some adolescents had parents who completed college or more (37.5%). Participants had a mean BMI of 24.5 (SEM ± 0.27), an average moderate-to-vigorous physical activity of 6.02 (SEM ± 0.1), and a mean depressive symptoms score of 10.8 (SEM ± 0.1). Most participants resided in low-poverty neighborhoods (56.4%).

Adolescents experiencing moderate/severe depressive symptoms had a mean age of 15.7 (SEM ± 0.1), being 63.8% females. The majority were NH White (55.5%), followed by NH Black (18.9%) and Hispanic (16.6%) adolescents. Most adolescents who experienced moderate/severe depressive symptoms were children of parent(s) who completed at least high school (37.9%). The majority of adolescents who had moderate/severe depressive symptoms were also from a neighborhood of low neighborhood poverty (50.7%). Strikingly, each PNSE score was lower in the mild and moderate/severe depressive symptoms groups than those of the minimal depressive symptoms group.

### Associations Between PNSE and Depressive Symptoms

Neighborhood safety, social cohesion, and contentedness were negatively associated with depressive symptoms (β = −1.14, 95% CI [−1.40, −0.89], β = −0.59, 95% CI [−0.81, −0.37], and β = −1.46, 95% CI [−1.72, −1.20], respectively, all *p* < 0.001; Table 2). Greater levels of perceived neighborhood safety, social cohesion, and contentedness were 22%, 17%, and 29% less likely to experience mild depressive symptoms, respectively, compared to those experiencing minimal symptoms (all *p* < 0.001). Greater levels of perceived neighborhood safety, social cohesion, and contentedness were 33%, 16%, and 38% less likely to experience moderate/severe depressive symptoms, respectively, compared to those experiencing minimal symptoms.

**Table 2.** Associations of Perceived Neighborhood Social Environment with Depressive Symptoms (*n* = 6,083)

	Continuous CES-D score^a^		Mild (16 ≤ CES-D <24) vs minimal (<16 referent)		Moderate/severe (CES-D ≥ 24) vs minimal (<16 referent)	
	Beta	95% CI	OR^b^	95% CI^c^	OR	95% CI
Safety	−1.14***	−1.40, −0.89	0.78***	0.73, 0.84	0.67***	0.61, 0.73
Social cohesion	−0.59***	−0.81, −0.37	0.83***	0.77, 0.90	0.84**	0.75, 0.93
Contentedness	−1.46***	−1.72, −1.20	0.71***	0.66, 0.77	0.62***	0.55, 0.70

* *p* < 0.05.

** *p* < 0.01.

*** *p* < 0.001.

^a^ CES-D, Center for Epidemiologic Studies Depression Scale.

^b^ OR, odds ratio.

^c^ 95% CI, 95% confidence interval.

### Analyses Stratified by Gender

Among male adolescents, neighborhood safety, social cohesion, and contentedness were negatively associated with depressive symptoms (β = −0.94, 95% CI [−1.31, −0.57], β = −0.53, 95% CI [−0.82, −0.24], and β = −1.14, 95% CI [−1.43, −0.85], respectively, all *p* < 0.001; Table 3). Among female adolescents, neighborhood safety, social cohesion, and contentedness were also negatively associated with depressive symptoms (all *p* < 0.001). These negative associations were more pronounced for females than those of male adolescents. For both male and female adolescents, there were negative associations between each PNSE and both mild and moderate/severe depressive symptoms compared to minimal depressive symptoms (Table 3).

**Table 3.** Gender-Specific Associations of Perceived Neighborhood Social Environment with Depressive Symptoms (*n* = 6,083)

	Continuous CES-D score^a^		Mild (16 ≤ CES-D < 24) vs minimal (<16 referent)		Moderate/severe (CES-D ≥ 24) vs minimal (<16 referent)	
	Beta	95% CI	OR^b^	95% CI^c^	OR	95% CI
Male (*n* = 2,941)						
Safety	−0.94***	−1.31, −0.57	0.74***	0.67, 0.83	0.68***	0.58, 0.78
Social cohesion	−0.53***	−0.82, −0.24	0.81**	0.71, 0.93	0.82*	0.68, 1.00
Contentedness	−1.14***	−1.43, −0.85	0.69***	0.61, 0.78	0.66***	0.55, 0.80
Female (*n* = 3,142)						
Safety	−1.37***	−1.77, −0.97	0.82***	0.74, 0.90	0.67***	0.59, 0.75
Social cohesion	−0.65***	−0.95, −0.36	0.84***	0.77, 0.92	0.84**	0.75, 0.95
Contentedness	−1.77***	−2.16, −1.37	0.72***	0.65, 0.79	0.60***	0.52, 0.69

* *p* < 0.05.

** *p* < 0.01.

*** *p* < 0.001.

^a^ CES-D, Center for Epidemiologic Studies Depression Scale.

^b^ OR, odds ratio.

^c^ 95% CI, 95% confidence interval.

### Analyses Stratified by Race/Ethnicity

Among NH White adolescents, neighborhood safety, social cohesion, and contentedness were negatively associated with depressive symptoms (β = −1.26, 95% CI [−1.68, −0.84], β = −0.76, 95% CI [−1.02, −0.49], and β = −1.64, 95% CI [−1.98, −1.31], respectively, all *p* < 0.001; Table 4). Among NH Black adolescents, neighborhood safety and contentedness were negatively associated with depressive symptoms (β = −1.11, 95% CI [−1.53–0.69] and β = −1.14, 95% CI [−1.68, −0.61], respectively, *p* < 0.001), but not for social cohesion. Similarly, among Hispanic adolescents, neighborhood safety and contentedness were negatively associated with depressive symptoms (β = −1.10, 95% CI [−1.66, −0.54] and β = −1.20, 95% CI [−1.79, −0.61], both *p* < 0.001, respectively), but not for social cohesion. Contentedness was negatively associated with depressive symptoms among NH Other adolescents (β = −1.25, 95% CI [−2.31, −0.20], *p* < 0.05). Similar associations for a continuous depressive symptoms outcome were observed for both mild and moderate/severe depressive symptoms outcomes.

**Table 4.** Race-Specific Associations of Perceived Neighborhood Social Environment with Depressive Symptoms (*n* = 6,083)

	Continuous CES-D score^a^		Mild (16 ≤ CES-D < 24) vs minimal (<16 referent)		Moderate/severe (CES-D ≥24) vs minimal (<16 referent)	
	Beta	95% CI	OR^b^	95% CI^c^	OR	95% CI
NH White (*n* = 3,558)						
Safety	−1.26***	−1.68, −0.84	0.73***	0.65, 0.82	0.66***	0.57, 0.77
Social cohesion	−0.76***	−1.02, −0.49	0.81***	0.73, 0.89	0.79**	0.69, 0.91
Contentedness	−1.64***	1.98, −1.31	0.67***	0.60, 0.74	0.60***	0.51, 0.70
NH Black (*n* = 1,455)						
Safety	−1.11***	−1.53, −0.69	0.81***	0.73, 0.89	0.66***	0.58, 0.76
Social cohesion	−0.58	−1.31, 0.14	0.84	0.67, 1.04	0.82	0.61, 1.10
Contentedness	−1.14***	−1.68, −0.61	0.75***	0.64, 0.88	0.66***	0.53, 0.83
Hispanic (*n* = 675)						
Safety	−1.10***	−1.66, −0.54	0.82*	0.69, 0.99	0.68***	0.57, 0.81
Social cohesion	0.02	−0.51, 0.55	0.96	0.81, 1.14	1.00	0.77, 1.28
Contentedness	−1.20***	−1.79, −0.61	0.85*	0.72, 0.99	0.63***	0.50, 0.80
Others (*n* = 395)						
Safety	−0.77	−1.70, 0.17	1.05	0.76, 1.46	0.63**	0.46, 0.84
Social cohesion	−0.41	−1.44, 0.62	0.78	0.57, 1.07	0.74	0.49, 1.11
Contentedness	−1.25*	−2.31, −0.20	0.68*	0.50, 0.92	0.51**	0.33, 0.78

* *p* < 0.05.

** *p* < 0.01.

*** *p* < 0.001.

^a^ CES-D, Center for Epidemiologic Studies Depression Scale.

^b^ OR, odds ratio.

^c^ 95% CI, 95% confidence interval.

## Discussion

Our study investigated the associations between PNSE factors (safety, social cohesion, and contentedness) and depressive symptoms using a nationally representative sample of adolescents from the Add Health cohort. As a secondary aim, these associations were varied by gender and race/ethnicity. Overall, PNSE factors were negatively associated with depressive symptoms. Compared to those reporting minimal depressive symptoms, adolescents perceiving higher PNSE factors had a lower likelihood of mild and moderate/severe depressive symptoms. These negative associations between PNSE and depressive symptoms appeared to be consistent with analyses stratified by gender. Specifically, a higher PNSE score for females was a protective factor against experiencing depressive symptoms than males. However, the mechanisms for these associations revealed complexity for certain race/ethnic groups in this sample of adolescents. Future research should consider replicating the relationships between environmental neighborhood determinants and depression among adolescents by various race/ethnic groups.

Consistent with previous studies, neighborhood safety, social cohesion, and contentedness were negatively associated with depressive symptoms.^12,14,24–26^ For example, a study focusing on adolescents using the California Health Interview Survey in 2011–2014 indicated that those perceiving higher neighborhood unsafety than those reporting safe neighborhoods were more likely to report serious psychological distress.^14^ In turn, a study using Canadian children indicated that those reporting higher neighborhood social cohesion had lower depressive symptoms.^24^ Similarly, one study using UK adolescents indicated that adolescents aged 18 years old perceiving lower neighborhood social cohesion compared to high cohesion were related to a greater likelihood of having high depressive symptoms.^26^ Moreover, this study indicated that mothers’ perceptions of neighborhood discord, stress, and social cohesion impacted depression among children.^26^ In our study, the finding of higher level of contentedness was linked to lower depressive symptoms which was also consistent with our results for safety and social cohesion. Findings from our study and others suggest that efforts toward improving conditions related to safety and cohesion (contentedness score) may effectively negate depressive symptoms.^13,14,24^

Gender-specific associations between PNSE and depressive symptoms revealed the important role of gender in this relationship. Greater perceived levels of neighborhood safety, social cohesion, and contentedness were protective factors for depressive symptoms for both genders. Yet, these associations were more pronounced among females. Other studies have also found gender differences in the relationship between perceived neighborhood environment and health; however, inconsistent associations by gender were found across studies.^15,24^ Like our findings, the study conducted among Canadian adolescents found that female adolescents experienced higher levels of depressive symptoms than those of their male counterparts.^24^ Moreover, female adolescents also had a more pronounced relationship between neighborhood characteristics and depression. The consistent findings between our study and the one conducted in Canada could be attributed to similar methods, including a similarly constructed adolescent depression score based on the self-reported CES-D.^24^ Contrastingly, a study that analyzed the association between perceived neighborhood safety and MDD among African American adolescents aged 13–17 found that perceived neighborhood safety was only associated with lower levels of MDD among African American male adolescents. The differences in findings can be attributed to many factors, however, including a smaller sample size (1,170 versus 6,083 participants) and racial and ethnic focus (only Black versus multiethnic adolescents).^15^ Therefore, the findings of this study strengthen the role of gender in the relationship between adolescents’ perceptions of the neighborhood environment and depression, which contribute further evidence to the gender-specific associations.

Race-specific associations between PNSE and depressive symptoms highlighted that race might also play an important role in this relationship. Our study found that participants who perceived higher neighborhood safety had lower depressive symptoms for NH White, NH Black, and Hispanic adolescents, but not NH Other groups, which was consistent with other studies among adults.^12,15,27–29^ To our knowledge, our study may be the first to examine whether the relationship between perceived neighborhood characteristics and adolescent depressive symptoms varied by racial and ethnic groups. Nevertheless, perceived neighborhood safety is an important factor in reducing depressive symptoms among Hispanic, Asian, Black, and White adults.^15,27–29^ One study examining this relationship among Hispanic populations employed a five-point Likert scale and five-item Geriatric Depression Scale to assess perceived neighborhood characteristics and depressive symptoms, respectively; a negative association between neighborhood safety and depressive symptoms was observed.^27^ Therefore, similar to our study, increased PNSE was inversely related to depressive symptoms among Hispanic individuals. Additionally, the study that explored the relationship between perceived neighborhood environments and depressive symptoms among Asian populations employed an adapted Quality of Korean Life Survey and a 10-item CES-D score.^28,30^ Again, these findings corroborate ours, highlighting the relationship between PNSE and depression for both Asian adolescents and adults. Moreover, a study found that increased perceptions of neighborhood characteristics in a predominantly White population living alone were a protective factor against the frequency of depressive symptoms.^29,31^ This study agrees with our findings, in that PNSE and depression are related among White individuals. Consistent with our study, another study examining the relationship between PNSE and depressive symptoms among a predominantly Black population found a negative relationship between higher levels of PNSE and depressive symptoms.^12^ Altogether, our findings highlight the importance of race in understanding the association of PNSE factors and depressive symptoms among adolescents.

### Strengths and Limitations

The strengths of this nationally representative study included a sizable study sample of adolescents of varying genders and races/ethnicities in the United States. Furthermore, this study investigated the role of gender and race/ethnic groups on the associations between PNSE factors and adolescent depressive symptoms. This critical component is missing from neighborhoods and mental health research. Classifying the severity of depressive symptoms as minimal, mild, and moderate/severe could provide a more accurate assessment for those with and without depression among adolescents.^32^

Despite these strengths, this study has several limitations. First, this study is cross-sectional. Therefore, causality cannot be inferred. Second, there is no robust data represented for Asian, Pacific Islander, or Native American/Indian adolescents within Add Health. These race and ethnic groups were all combined in the “NH Other” category. Furthermore, a study examining the relationship between perceived neighborhood safety and adolescent depression between African American and Caribbean Black adolescents found that ethnicity played a role in this relationship, highlighting how studying race alone may not fully portray this relationship.^15^ Future studies should disentangle the effects of race and ethnicity on the examined associations. Finally, this study uses data from Wave 1 of Add Health, which was collected from 1994 to 1995. Therefore, our results might not be generalizable to adolescents today. Nevertheless, Add Health provides unique insight into adolescent outcomes, as it is one of the largest nationally representative databases on various aspects of adolescent health. Furthermore, despite this limitation, our study also provides retrospective insight into longstanding gender and racial/ethnic disparities underlying the association between neighborhood contexts and adolescent depression.

## Conclusion

Using a nationally representative sample of U.S. adolescents, we investigated the associations between PNSE and depressive symptoms. Overall, our study found that higher PNSE (i.e., safety, social cohesion, contentedness) was associated with lower depressive symptoms among adolescents. Gender-specific associations were similar, with stronger results in females. Additionally, race-specific associations indicated that heightened levels of PNSE factors were related to lower adolescent depressive symptoms among all races. Therefore, our study revealed intricate dynamics between depression and perceived neighborhood environment of U.S. adolescents living when considering gender and race/ethnicity.

## Data Availability

The data that support the findings of this study are openly available in the Interuniversity Consortium for Political and Social Research (ICPSR) at https://www.icpsr.umich.edu/web/pages, reference number (ICPSR 21600).

## Abbreviations Used

95% CI: 95% confidence interval
Add Health: National Longitudinal Study of Adolescent to Add Health
BMI: Body mass index
CES-D: Center for Epidemiologic Studies Depression Scale
IRB: Institutional Review Board
MDD: Major depressive disorder
MDE: Major depressive episodes
MVPA: Moderate-to-vigorous physical activity
N: number
NH: Non-Hispanic
OR: Odds ratio
PA: Physical activity
PNSE: Perceived neighborhood social environment
SEM: Standard error of mean
